# FOXP1 is differentially active during development of murine vasopressin and oxytocin magnocellular neurons

**DOI:** 10.1016/j.isci.2026.115604

**Published:** 2026-04-04

**Authors:** Jari B. Berkhout, Sophie Trender, Quirin Krabichler, Yuval Podpecan, Felix Franke, Tim Schubert, Peter Burbach, Valery Grinevich, Roger Adan, Henning Fröhlich, Ferdinand Althammer, Onno C. Meijer, Ahmed Mahfouz

**Affiliations:** 1 Department of Internal Medicine, Division Endocrinology, Leiden University Medical Center, Leiden, the Netherlands; 2 Department of Human Genetics, Leiden University Medical Center, Leiden, the Netherlands; 3 Institute of Human Genetics, Heidelberg University Hospital, Heidelberg, Germany; 4 Department of Neuropeptide Research in Psychiatry, Central Institute of Mental Health, University of Heidelberg, Mannheim, Germany; 5 Department of Translational Neuroscience, Brain Center Rudolf Magnus, University Medical Center Utrecht, Utrecht, the Netherlands; 6 Department of Intelligent Systems, Division Pattern Recognition and Bioinformatics, Technical University Delft, Delft, the Netherlands

**Keywords:** Molecular physiology, Neuroscience, Developmental biology

## Abstract

Hypothalamic arginine vasopressin (AVP) and oxytocin (OXT) magnocellular neurons (MCNs), share a developmental lineage. The transcription factors driving specification are yet unknown. Using gene regulatory network analysis on published single-cell RNA-sequencing data of the developing mouse hypothalamus, we identified RORA, EBF3, FOXP1, FOXP2, and BCL11B as candidate transcription factors for differential MCN specification. We modeled developmental gene expression dynamics using computational cell fate mapping, revealing enrichment of EBF3 and BCL11B in the *Avp* lineage, and FOXP1 and FOXP2 in the *Oxt* lineage. *In silico* analysis of *Avp* and *Oxt* promoters predicted a binding site for FOXP1 and FOXP2, and *an in vitro reporter* assay identified regulation on both *Avp* and *Oxt* genomic promoters. Finally, heterozygous FOXP1 knockout mice exhibited a significant reduction in AVP and OXT neuron abundance, with OXT neurons disproportionally affected. We conclude that FOXP1 participates in MCN development, while being differentially active in OXT MCNs relative to AVP MCNs.

## Highlights

• Computational analysis reveals candidate TFs for AVP and OXT neuron differentiation
• EBF3/BCL11B and FOXP1/FOXP2 are enriched in *Avp* and *Oxt* neurons, respectively
• FOXP1 and FOXP2 regulate *Avp* and *Oxt* gene expression *in vitro*
• Reduced abundance of AVP and OXT neurons and reduced OXT/AVP ratio in FOXP1^+/−^ mice

## Graphical abstract

**Figure:**
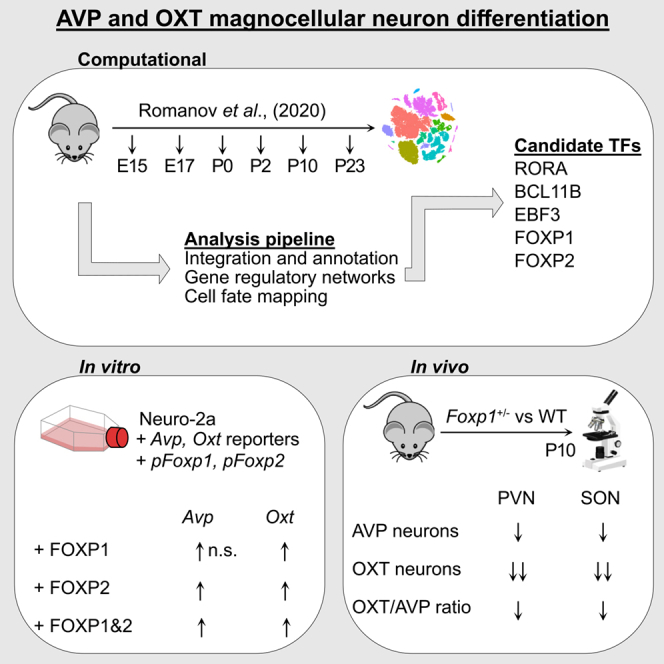

## Introduction

A long-standing open question in neuroendocrinology is the origin of the transcriptional differences between the hypothalamic magnocellular neuron (MCN) types. These neurons are located in the hypothalamic paraventricular nucleus (PVN) and supraoptic nucleus (SON), where they produce the neuropeptides arginine vasopressin (AVP) and oxytocin (OXT). Structurally, AVP and OXT are closely related nonapeptides, differing at only two amino acids. The genes encoding for AVP and OXT are only 10 kb apart on the human genome, and are thought to be the result of a gene duplication event around the origin of vertebrates.^1^^,^^2^ Despite their evolutionary relatedness, AVP and OXT differ in hormonal function: AVP regulates arterial blood pressure and water homeostasis,^3^ while the best known function of OXT is the induction of parturition^4^ and lactation.^5^ As systemic hormones, AVP and OXT are released from MCNs projections to the posterior pituitary. Despite their similarities, AVP and OXT are predominantly produced in neuropeptide-specific MCNs.^6^

AVP and OXT MCNs are clearly distinct, yet similar in some respects. A common feature is the known developmental trajectories of both lineages. Development of both AVP and OXT MCNs necessitates the expression of transcription factors (TFs) OTP, SIM1, ARNT2 and POU3F2.^7^^,^^8^^,^^9^^,^^10^^,^^11^^,^^12^ Also, it has been known for some time that AVP and OXT MCNs express mRNA for their counterpart peptides, though at levels differing by orders of magnitude.^13^^,^^14^ Recently, we corroborated these findings with a single-cell transcriptomic atlas of the PVN, and found the AVP and OXT MCNs to be highly transcriptionally correlated, yet also distinct.^15^

The evolutionary relatedness, high level of transcriptional similarity, and largely overlapping ontogeny imply a common developmental progenitor. Yet, the presumed TF(s) involved in the divergence of the AVP and OXT lineages have not been described yet. While several candidate TFs have been proposed to potentially drive the differential expression of *AVP* and *OXT*,^16^ none have been conclusively established to do so. In this work, we aim to answer this question using publicly available single-cell transcriptomics data, an *in vitro* reporter assay, and to validate one of the identified candidate TFs *in vivo*.

## Results

### Publicly available single-cell RNAseq reveals all developmental stages of MCNs

**Figure 1:**
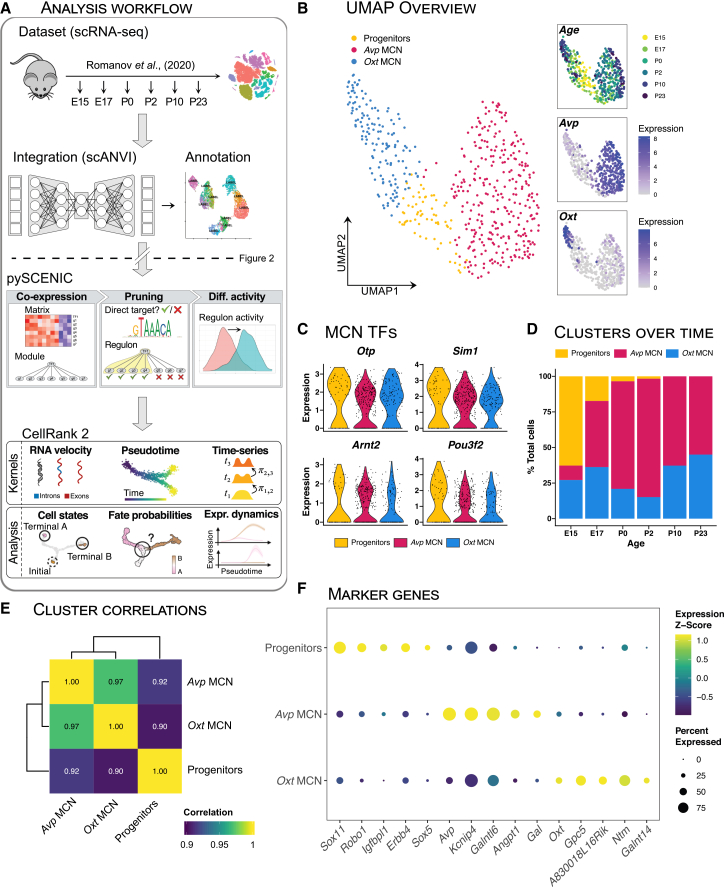
Developmental single-cell RNA-seq (scRNA-seq) of magnocellular neurons (MCNs) reveals three distinct clusters (A) Workflow of the analysis presented in this Figures 1 and 2. The developmental mouse dataset from Romanov et al. (2020)^17^ was integrated with scANVI,^18^ and then clustered and annotated. Subsequently, the pySCENIC^19^ pipeline was performed to infer gene regulatory networks. Finally, the CellRank 2 pipeline^20^ was applied to infer cell fate probabilities. (B) (Left) Uniform manifold approximation and projection (UMAP) showing the clusters as annotated after integration. (Right) UMAPs with coloring represent sampling timepoints (age), *Avp* expression, and *Oxt* expression. (C) Violin plots of the expression levels for known MCN-expressed transcription factors (TFs) *Otp, Sim1, Arnt2,* and *Pou3f2*. (D) Stacked bar plots of the proportion of cells annotated as each cluster over the different sampling timepoints. (E) Heatmap of the correlation coefficients between pseudobulk expression profiles for each cluster in the dataset. All clusters are highly correlated; coloring is scaled to show mutual differences in correlations. (F) Dotplot of the normalized expression levels for cluster-enriched gene markers. Coloring represents Z-scaled expression levels, and dot size represents the percentage expression of a marker within the respective cluster.

We used the developmental single-cell dataset from Romanov et al. (2020),^17^ comprising mouse hypothalamus tissue at several developmental timepoints: embryonic days 15 and 17 (E15, E17), and post-natal days 0, 2, 10, and 23 (P0, P2, P10, P23; Figure 1A). A subset of the dataset was taken based on annotations as defined by Romanov et al. (2020),^17^ to only include MCNs. The subset was then integrated to eliminate internal batch effects, and subsequently clustered based on the integrated embedding.

We identified three clusters of MCNs: *Avp* MCNs and *Oxt* MCNs, and a third MCN cluster likely to be progenitors (Figure 1B). The *Avp* and *Oxt* MCNs strongly expressed *Avp* and *Oxt*, respectively, while the progenitors did not express either neuropeptide (Figure 1B). All three clusters expressed high levels of the currently known MCN TFs *Otp, Sim1, Arnt2,* and *Pou3f2* (Figure 1C). The progenitor cluster was most abundant at the earlier time points, diminishing in abundance over time (Figure 1D). All clusters were highly correlated to each other, with the progenitor cluster being relatively most distinct from both *Avp* MCNs and *Oxt* MCNs (Figure 1E). Progenitors were found to express high relative levels of *Sox11, Robo1, Igfbpl1, Erbb4,* and *Sox5*. *Avp* MCNs specifically expressed *Avp, Kcnip4, Galntl6, Angpt1,* and *Gal.* Finally, *Oxt* MCNs specifically expressed *Oxt, Gpc5, A830018L16Rik, Ntm,* and *Galnt14* (Figure 1F).

### RORA, EBF3, FOXP1, FOXP2, and BCL11B are candidate TFs for diverging differentiation

**Figure 2:**
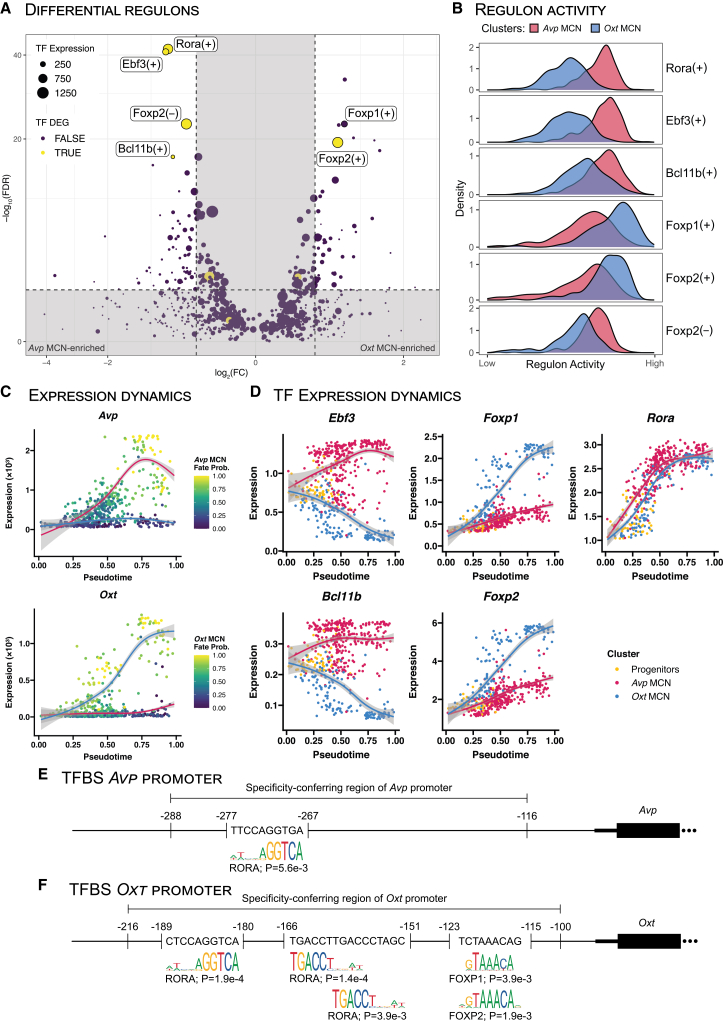
Rora, Ebf3, Foxp1, Foxp2, and Bcl11b are candidate TFs for diverging *Avp* vs. *Oxt* MCN differentiation (A) Volcano plot with differential regulons activity scores in *Avp* MCNs (left) versus *Oxt* MCNs (right). Activating regulons are denoted with a plus sign, repressive regulons with a minus sign. Dot size represents the gene expression levels of the TF defining the regulon. Dot colors represent whether the gene expression of the TF defining the regulon is differential between clusters. (B) Density plots of the regulon activity scores in the *Avp* and *Oxt* MCN clusters, aggregated over all time points. (C) Denoised gene expression dynamics of *Avp* and *Oxt* over pseudotime. Dot colors show cell fate probabilities for differentiation toward the *Avp*^+^ fate (upper) or *Oxt*^+^ fate (lower). Curves are fitted using a generalized additive model weighted by the fate probability odds ratios. Curve colors correspond to the coloring used in Figure 1B. (D) Denoised gene expression dynamics of *Rora*, *Ebf3*, *Foxp1*, *Foxp2*, and *Bcl11b* over pseudotime. Dot colors reflect clusters assigned after integration. Curves are fitted using a generalized additive model weighted by the fate probability odds ratios. Dot and curve colors correspond to the coloring used in Figure 1B. (E) Schematic representation of the mouse *Avp* locus, with a focus on the specificity-conferring region (SCR) as defined by Ponzio et al. (2012).^21^ Predicted TFBSs are shown, including coordinates and the aligned JASPAR motifs. Coordinate placement not to scale. (F) Schematic representation of the mouse *Oxt* locus, with a focus on the specificity-conferring region (SCR) as defined by Fields et al. (2012).^22^ Predicted TFBSs are shown, including coordinates and the aligned JASPAR motifs. Coordinate placement not to scale.

Next, we used pySCENIC^19^ to identify candidate gene regulatory processes underlying Avp-Oxt differentiation. pySCENIC infers gene regulatory networks (GRNs) centered around TFs—also known as regulons –through co-expression analysis paired with TF binding site (TFBS) enrichment analysis (Figure 1A). A regulon can be based on either positively correlating components or negatively correlating components, resulting in activating and repressive regulons, respectively. An activity score is then assigned for each regulon in each cell, which was used to calculate differentially active regulons between the *Avp* and *Oxt* clusters (Figure 2A). To narrow down our selection, regulons were filtered out if the associated TF was not a significantly differentially expressed gene (DEG) between the *Avp* and *Oxt* clusters.

With these criteria, the activating regulons Rora(+), Ebf3(+), Foxp2(+) and Bcl11b(+) were included, as well as the repressive regulon Foxp2(−). In addition, the differentially active regulon Foxp1(+) was included despite the lack of differential *Foxp1* gene expression. Although not differentially expressed between *Avp and Oxt clusters*, *Foxp1* could be biologically relevant and might in fact drive the observed differential expression of the Foxp2 regulons through its well-described ability for heterodimerization with *Foxp2*.^23^^,^^24^ From the selected regulons, Rora(+), Ebf3(+), Bcl11b(+), and Foxp2(−) were found enriched in *Avp* MCNs, while Foxp1(+) and Foxp2(+) were enriched in *Oxt* MCNs (Figure 2B).

Of note, this differential activity analysis does not account for temporal dynamics. To interrogate the temporal dynamics of the resulting regulons, we applied CellRank2, utilizing RNA velocity,^25^ pseudotime,^26^ and optimal-transport analysis^27^ to infer cell fate probabilities (see STAR Methods). With these cell fate probabilities, we then visualized the expression dynamics of genes throughout pseudotime (Figure 2C). Analyzing the expression dynamics for *Rora, Ebf3, Foxp1, Foxp2,* and *Bcl11b* revealed the expression pattern for *Rora* to be highly similar between *Avp* and *Oxt* clusters (Figure 2D)*.* Both *Ebf3* and *Bcl11b* expression increase in *Avp* MCNs and decrease in *Oxt* MCNs (Figure 2D). Finally, the expression patterns for *Foxp1* and *Foxp2* were found to be highly similar to each other, both increasing in *Avp* MCNs as well as *Oxt* MCNs, albeit at a considerably faster rate in the latter (Figure 2D). A key difference is the overall expression levels of these factors, which were higher for *Foxp2* than for *Foxp1.* This lower *Foxp1* expression and subsequent smaller absolute difference between populations may also explain the observed lack of significant *Foxp1* expression difference between *Avp* and *Oxt* MCNs. Notably, the observed expression patterns for both *Foxp1* and *Foxp2* are very similar to the expression pattern for *Oxt.*

We then investigated if binding sites for the identified TFs exist within the *Avp* or *Oxt* promoters to directly regulate these neuropeptides. Previous work on the mouse *Avp* and *Oxt* promoters defined regions in both promoters that are necessary for cell-type specific expression.^16^^,^^21^^,^^22^ Within these specificity-conferring regions (SCRs), we analyzed the presence of TFBSs for candidate TFs (RORA, EBF3, FOXP1, FOXP2, and BCL11B) using their corresponding motifs from JASPAR.^28^ The *Avp* SCR contained a single TFBS for RORA (Figure 2E). The *Oxt* SCR contained several TFBSs for RORA, and a single TFBS for both FOXP1 and FOXP2 (Figure 2F). These findings align with Gainer (2012),^16^ who reported predicted TFBSs in the *Oxt* SCR for RORA and FOXO1, a TF with a motif highly similar to both FOXP1 and FOXP2.

### *In silico* and *in vitro* analyses further substantiate a role for FOXP1 and FOXP2

**Figure 3:**
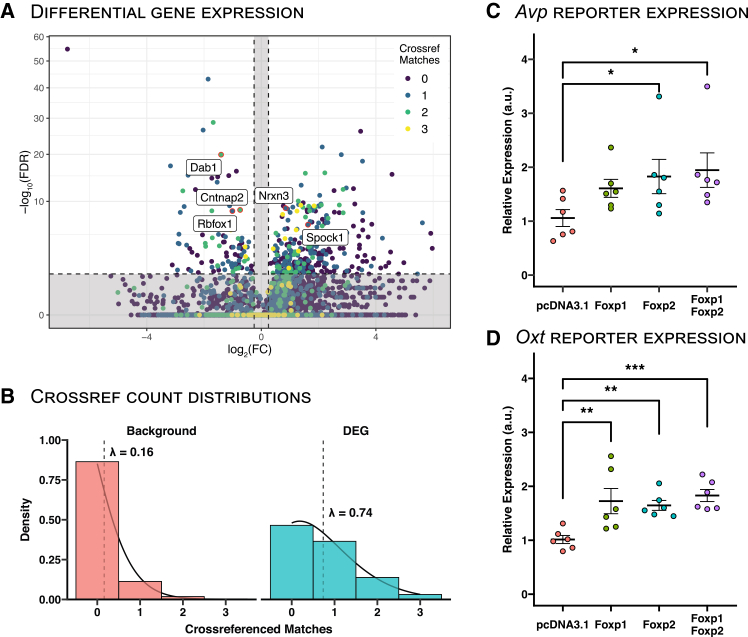
FOXP1 and FOXP2 target genes are enriched in *Avp* versus *Oxt* differentially expressed genes (DEGs), and increase *Avp* and *Oxt* reporter activity *in vitro* (A) Volcano plot with differential gene expression between in *Avp* MCNs (left) versus *Oxt* MCNs (right). Dots are colored by the number of cross-referenced matches to previously reported FOXP1 and FOXP2 DEGs. Several known FOXP1 and FOXP2 target genes have been highlighted with a text label. (B) Distribution of cross-referenced matches in the genomic background (left) versus DEGs (right). Dotted line denotes the fitted lambda of the Poisson GLM, and the solid line denotes the associated fitted Poisson distribution. (C) Relative gene expression (a.u.) of the *Avp* reporter gene for all transfection combinations of FOXP1 and FOXP2. Crossbar and error bars denote mean ± SE. ∗*p* < 0.05, ∗∗*p* < 0.01, and ∗∗∗*p* < 0.001. (D) Relative gene expression (a.u.) of the *Oxt* reporter gene for all transfection combinations of FOXP1 and FOXP2. Crossbar and error bars denote mean ± SE. ∗*p* < 0.05, ∗∗*p* < 0.01, and ∗∗∗*p* < 0.001. Asterisks indicate statistically significant differences as determined by a Tukey’s honest significant differences test (∗*p* < 0.05, ∗∗*p* < 0.01, and ∗∗∗*p* < 0.001).

Our results so far strongly suggest that FOXP1 and/or FOXP2 play a role in Avp-Oxt MCN differentiation by regulating *Oxt* but not *Avp* MCNs. To further validate the computational predictions of FOXP1 and FOXP2 activity derived from pySCENIC, we cross-referenced DEGs between *Avp* and *Oxt* MCNs with established FOXP1- and FOXP2-regulated genes. To achieve this, we leveraged published bulk and single-cell RNA-seq datasets to identify significantly up- and downregulated genes in FOXP1 and FOXP2 knockout models across various neuronal contexts.^29^^,^^30^^,^^31^^,^^32^ Among our DEGs, we identified several previously reported FOXP targets, including *Cntnap2*,^33^^,^^34^ *Rbfox1*^35^*, Nrxn3*,^31^^,^^32^^,^^36^^,^^37^ *Spock1*,^33^^,^^36^ and *Dab1*^38^ (Figure 3A). Overall, we observed a significant 4.6-fold enrichment of known FOXP-regulated genes within our DEGs, compared to the genomic background (Figure 3B; Poisson GLM; β = 1.52, z = 25.42, *p* < 2.2 × 10^−16^).

Following up on these *in silico* findings, we investigated whether FOXP1 or FOXP2 can directly regulate *Avp* or *Oxt,* using an *in vitro* dual-reporter assay in Neuro-2a cells. To test the effects of FOXP1 and FOXP2 transfection on *Avp* reporter expression, a two-way ANOVA was conducted (Figure 3C). A significant main effect was found for FOXP2 (F_1,20_ = 6.68, *p* = 0.018) but not FOXP1 (F_1,20_ = 3.73, *p* = 0.068) or the interaction effect (F_1,20_ = 1.94, *p* = 0.179). Subsequently, post-hoc analyses revealed a significant difference between pcDNA3.1 control (*n* = 6) and FOXP1/FOXP2 (*n* = 6; *p* = 0.022) co-transfection and between pcDNA3.1 and FOXP2 (*n* = 6; *p* = 0.049). No significant effect was observed between pcDNA3.1 and FOXP1 (*n* = 6; *p* = 0.12) transfection.

The same analysis was performed for the *Oxt* reporter assay (Figure 3D). Here, significant main effects were observed for FOXP1 (F_1,20_ = 12.9, *p* = 0.0018), FOXP2 (F_1,20_ = 12.0, *p* = 0.0025), and the interaction effect (F_1,20_ = 5.6, *p* = 0.028). Post-hoc analysis revealed significant differences between the pcDNA3.1 control and FOXP1 (*p* = 0.0022), FOXP2 (*p* = 0.0028), and FOXP1/FOXP2 (*p* = 3.9 × 10^−4^) co-transfections. In summary, significant effects were observed for FOXP1 on *Avp* reporter activity and for FOXP1 and FOXP2 on *Oxt* reporter activity. It should be noted that effect sizes were roughly equal between *Avp* reporter and *Oxt* reporter expression changes. While not reaching significance for some *Avp* reporter comparisons, conclusions regarding (lack of) effect should be made cautiously.

### *Foxp1*^+/−^ mice have disproportionally fewer OXT neurons than wild-type littermates at P10

The enrichment of previously reported FOXP1 and FOXP2 targets in *Avp*-*Oxt* MCN DEGs and the regulation of both promoters further substantiated our hypothesis that FOXP1 and FOXP2 have a developmental role in these neurons. To further investigate this in a physiologically relevant context, we opted to analyze whole hypothalami of *Foxp1* heterozygous knockout mice (*Foxp1*^*+/−*^), the only model available to us. Homozygous knockouts are embryonically lethal,^39^ and *Foxp1*^+/−^ mice have previously been used to study the effects of *Foxp1* haploinsufficiency in the brain.^30^^,^^40^ Accordingly, PVN- and SON-containing brain sections from P10 male *Foxp1*^+/−^ mice (*n* = 7) and wild-type (WT; *n* = 7) littermates were stained for AVP and OXT, and subsequently imaged using confocal microscopy. The resulting 3D images were fully quantified to measure AVP and OXT neuron abundance, as previously described.^41^ We hypothesized that the heterozygous knockout would induce a selective reduction in OXT neurons compared to WT mice.

**Figure 4:**
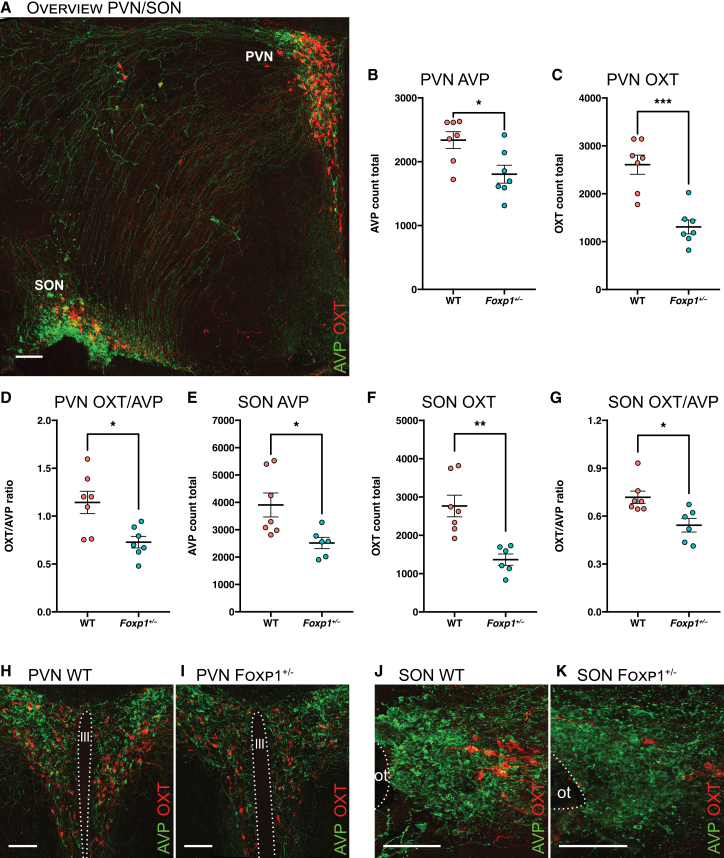
*Foxp1*^+/−^ mice exhibit disproportionally fewer OXT neurons in both the paraventricular nucleus (PVN) and supraoptic nucleus (SON) at P10 (A) Overview image of the mouse hypothalamus at P10, stained for AVP (green) and OXT (red). Scale bars, 100 μm. (B) Total observed OXT neurons in the whole PVN for *Foxp1*^+/−^ mice compared to wild-type (WT) littermates. Crossbar and error bars denote mean ± SE. ∗*p* < 0.05, ∗∗*p* < 0.01, and ∗∗∗*p* < 0.001. (C) Total observed AVP neurons in the whole PVN for *Foxp1*^+/−^ mice compared to WT littermates. Crossbar and error bars denote mean ± SE. ∗*p* < 0.05, ∗∗*p* < 0.01, and ∗∗∗*p* < 0.001. (D) Ratio of observed OXT/AVP neurons in the whole PVN for *Foxp1*^+/−^ mice compared to WT littermates. Crossbar and error bars denote mean ± SE. ∗*p* < 0.05, ∗∗*p* < 0.01, and ∗∗∗*p* < 0.001. (E) Total observed OXT neurons in the whole SON for *Foxp1*^+/−^ mice compared to WT littermates. Crossbar and error bars denote mean ± SE. ∗*p* < 0.05, ∗∗*p* < 0.01, and ∗∗∗*p* < 0.001. (F) Total observed AVP neurons in the whole SON for *Foxp1*^+/−^ mice compared to WT littermates. Crossbar and error bars denote mean ± SE. ∗*p* < 0.05, ∗∗*p* < 0.01, and ∗∗∗*p* < 0.001. (G) Ratio of observed OXT/AVP neurons in the whole SON for *Foxp1*^+/−^ mice compared to WT littermates. Crossbar and error bars denote mean ± SE. ∗*p* < 0.05, ∗∗*p* < 0.01, and ∗∗∗*p* < 0.001. (H) Representative image of a WT PVN, stained for AVP (green) and OXT (red). Scale bars, 100 μm. (I) Representative image of a *Foxp1*^+/−^ PVN, stained for AVP (green) and OXT (red). Scale bars, 100 μm. (J) Representative image of a WT SON, stained for AVP (green) and OXT (red). Scale bars, 100 μm. (K) Representative image of a *Foxp1*^+/−^ SON, stained for AVP (green) and OXT (red). Scale bars, 100 μm. Asterisks indicate statistically significant differences as determined by a Welch’s *t* test (∗*p* < 0.05, ∗∗*p* < 0.01, and ∗∗∗*p* < 0.001).

In the PVNs of *Foxp1*^+/−^ mice, fewer OXT neurons were observed than in WT (Figure 4B; *t* = 5.3; *p* = 2.8 × 10^−4^). A more modest decrease was observed for AVP neurons (Figure 4C; *t* = 2.8; *p* = 0.017). Further, the ratio between OXT and AVP neurons was decreased significantly in the *Foxp1*^+/−^ mice (Figure 4D; *t* = 3.2; *p* = 0.008), indicating that the partial loss of FOXP1 disproportionally affected OXT neurons. The same pattern was observed for the SON. Here again, the strongest decrease was observed in OXT neurons (Figure 4E; *t* = 4.4; *p* = 0.002), and a smaller decrease was observed in AVP neurons (Figure 4F; *t* = 2.9; *p* = 0.020). Similarly, the ratio between OXT and AVP neurons was decreased significantly in the SON as well (Figure 4G; *t* = 3.0; *p* = 0.015).

## Discussion

In this study, we investigated the divergent differentiation of AVP and OXT MCNs using publicly available single-cell data of the developing mouse hypothalamus. Previous research on the differences between AVP and OXT MCNs has focused mainly on the neuron-specific expression of the respective neuropeptides, through the investigation of specificity-conferring promoter regions, rather than on the TF(s) responsible for the broader divergent differentiation. Instead, we leveraged single-cell computational analysis to investigate differentially active TFs between AVP and OXT MCN transcriptomes. Our analysis yielded five candidate TFs that may mediate the divergent differentiation of these neurons. For all candidates but RORA, we subsequently found diverging developmental gene expression patterns, supportive of the validity of the GRN analysis.

Our analysis of the SCRs of the *Avp* and *Oxt* promoters identified only RORA as a TF binding the *Avp* promoter, and three candidates (RORA, FOXP1, FOXP2) were predicted to bind the *Oxt* promoter. Interestingly, RORA was implicated here with several TFBSs in both promoters. However, these findings lack interpretability due to earlier conflicting findings and reports. The expression dynamics of *Rora* between populations were virtually identical, unlike the regulon activity and gene expression, which were found enriched in *Avp* neurons. These findings are complicated by previous work, experimentally showing RORA to drive the mouse *Oxt* promoter,^42^ despite its enriched expression in *Avp* MCNs. In addition, RORA expression does not seem to be evolutionarily conserved. In rats, previous work has found *Rora* to be enriched in *Oxt* neurons,^43^ in contrast to our findings in mice. Due to this ambiguity surrounding the role of RORA, we focused our analysis on FOXP1 and FOXP2, instead.

We then performed a FOXP1- and FOXP2-targeted *in silico* analysis, by cross-referencing *Avp-Oxt* DEGs with previously identified FOXP1- and FOXP2-regulated genes.^29^^,^^30^^,^^31^^,^^32^ We discovered an enrichment of cross-referenced matches in the DEGs, compared to the genomic background. This substantiated our supposition of differential FOXP1 and FOXP2 activity. Next, we performed an *in vitro* reporter assay to determine if FOXP1 and FOXP2 directly regulate *Avp* and *Oxt*. Here, we observed an effect of both FOXP1 and FOXP2 on *Oxt* promoter activity, and an effect of FOXP2 on *Avp* promoter activity. Notably, while the effect of FOXP1 on *Avp* promoter activity did not reach significance, the observed effect size was equivalent to the effect on the *Oxt* promoter, but with increased variance. Regardless, the observed effect on the *Avp* promoter was unexpected, considering the lack of a motif in the promoter SCR. If these results reflect the physiological environment of MCNs, they suggest a complex regulatory landscape. Specifically, the high FOXP1 and FOXP2 activity in *Oxt* MCNs would theoretically drive both *Oxt* and *Avp* expression; therefore, an independent inhibitory mechanism would be required to suppress *Avp* in these cells. Conversely, because FOXP1 and FOXP2 expression is limited in *Avp* MCNs, a cell-type-specific co-activator must be necessary to drive *Avp* expression.

Finally, we used the *Foxp1*^+/−^ mouse model, the only model available to us. In this mouse line, we investigated whether *Foxp1* haploinsufficiency affects AVP and OXT neuron abundance in the PVN and SON. As this study focused solely on MCNs, it should be noted that the PVN contains both MCNs and parvocellular neurons (PCNs). Thus, the decreased OXT neuron abundance in the PVN may also be attributed to the PCNs present there, and this effect cannot be deconvolved with our current data. Nevertheless, the SON does contain only MCNs, and these results do conclusively determine the effect of *Foxp1* haploinsufficiency on MCNs.

We observed that the partial loss of *Foxp1* expression decreased the abundance of both AVP and OXT neurons at P10. The effect on the AVP neurons was again unexpected, though not without a plausible explanation. Our modeled expression dynamics show limited *Foxp1* and *Foxp2* expression in *Avp* neurons, not a complete lack of expression. As such, these factors may, at these lower concentrations, still affect *Avp* transcriptional activity and AVP MCN development, albeit less impactfully. Importantly, the OXT neuron abundance was disproportionally decreased at P10. This preferential decrease is consistent with our prediction that there is a previously unrecognized role for FOXP1 in OXT MCNs, although its exact role in MCNs appears more nuanced than a simple binary on/off model would suggest.

A previously uncharacterized developmental role fits well in the current knowledge on FOXP1, which has been associated with developmental functional expression in various neuronal tissues. For instance, in the neocortex, FOXP1 regulates apical radial glia self-renewal and the timing of neurogenesis.^44^ Consequently, loss of FOXP1 here leads to precocious cell cycle exit, progenitor depletion, and defects in both laminar fate acquisition and neuronal migration.^32^ In the striatum, FOXP1 is essential for the specification and maintenance of distinct medium spiny neuron populations, particularly those of the indirect pathway. Its loss results in a significant reduction in this population’s numbers and disrupts the striatal structural organization.^29^

While previous reports of FOXP1 in the context of the PVN and MCNs are scarce, it has been noted before—including by us—that FOXP1 is a specific marker of MCNs, in contrast to the *Avp-* and *Oxt*-expressing PCNs.^15^^,^^45^ Within this adult MCN context, we previously encountered FOXP gene expression profiles in *Oxt* MCNs and *Avp* MCNs similar to the present study, except for osmotically stimulated *Avp* MCNs.^15^ This distinct *Avp* MCN subpopulation expressed increased levels of *Foxp1* and *Foxp2* compared to all other MCNs, and increased *Avp and Oxt* compared to baseline *Avp* MCNs. Considering our findings in the current work, this *Avp* MCN population could be of interest regarding a role for FOXP1 and FOXP2 beyond pre- and postnatal early development.

Although FOXP1 and OXT have not been functionally related before in the literature, some notable phenotypic parallels can be found that connect the two. One of these is the mouse ultrasonic vocalizations (USVs) that are emitted by pups in various situations, including isolation from the nest. In mice that (partially) lack *Foxp1*, *Oxt* or its receptor (*Oxtr*), the number of USVs has been shown to decrease markedly.^30^^,^^46^^,^^47^^,^^48^ Another parallel is autism spectrum disorder (ASD), a neurodevelopmental condition characterized by social deficits, repetitive behaviors, and sensory processing differences.^49^ In mice, the knockouts of *Oxt* and *Oxtr* lead to social deficits,^46^^,^^50^ while *Foxp1* knockout mice display both social deficits and repetitive behaviors.^51^ In humans, ASD has been linked to lower plasma OXT levels and variants of the OXT receptor.^52^^,^^53^ Moreover, individuals with FOXP1 syndrome, harboring a deleterious variant in the *FOXP1* gene, often present with ASD as one of its various features.^54^ Until now, these effects were often thought to occur due to the important role of FOXP1 in striatal medium spiny neurons.^30^ However, given our present findings, these phenotypic similarities may also partially be explained through the effect of FOXP1 variants on OXT MCNs.

Some of the other TFs of interest have previously reportedly been linked to FOXP1 and FOXP2. For instance, *Foxp1* knockdown has been shown to reduce BCL11B expression during *in utero* cortical development.^55^ Conversely, striatal FOXP1 expression was reduced after *Bcl11b* knockout.^56^ Further, in a *Foxp1* conditional knockout model, cortical *Rora* expression was shown to be lost, while in that study *Bcl11b* seemed unaffected.^32^ Finally, POU3F2, the canonical MCN TF highly expressed in both *Avp* and *Oxt* MCNs has been linked to FOXP2 through an intronic regulatory element that can drive *Foxp2* expression in vertebrates.^57^ This context hints at the possibility of a TF network regulating differentiation, where the total transcriptional profile may be governed by the weighted contributions of the various TFs within the network. This could explain the observed effects in AVP MCNs: While FOXP1’s regulatory weight is lower in AVP cells than in OXT cells, it remains sufficient to elicit a detectable phenotypic effect.

Concluding, for follow-up research, the precise mechanism of action underlying reduced *Avp* and *Oxt* MCN abundance remains to be fully explained. Additionally, other identified TFs could prove viable candidates to investigate, possibly as part of a TF network governing AVPergic and OXTergic differentiation. Regardless, while there are many potential additional avenues for follow-up work to be tested, our data unequivocally shows that FOXP1 has a role in MCN development, and that its reduced presence disproportionally affects OXT neurons. This finding might contribute to explaining social aspects of FOXP1 syndrome and could unlock novel treatment strategies that target OXT system deficiencies.

### Limitations of the study

This study does present some limitations. First, based on our current data, we cannot determine conclusively if the deficient OXT MCN abundance in *Foxp1*^+/−^ mice should be viewed as impaired specification, developmental delay, or a failure in post-developmental maintenance resulting in apoptosis. While the underlying cellular kinetics differ, the ultimate functional implications for the oxytocinergic system remain largely equivalent. Even delayed development could plausibly result in social deficits, due to the critical postnatal window for OXT system development.^58^ This phenomenon can be observed in two mouse models with OXT dysfunction. In mice lacking the *Magel2* gene, a series of early-postnatal OXT injections rescued social and learning behavior in adulthood.^59^ Similarly, in mice lacking the *Cntnap2* gene, postnatal OXT injections rescued the social behavioral phenotype in adults.^60^ Interestingly, *Cntnap2* is a known target gene of FOXP1 and FOXP2.^34^^,^^61^ These results show that the postnatal time period is crucial for normal development of OXT signaling, and *Foxp1*^+/−^ mice might be similarly affected, even if OXT neuron development is only delayed.

Second, we used a whole-body mutant mouse line, and not a PVN- or MCN-specific knockout. Thus, the decreased neuron abundance observed in the mutant mice may have arisen through interactions with other brain regions or peripheral tissues. However, as both our computational analysis and *in vitro* analysis independently suggest a role for FOXP1, we estimate this alternative explanation to be unlikely. Nevertheless, a PVN- or MCN-specific knockout would be beneficial for follow-up beyond this limitation. It would enable the investigation of a homozygous knockout, without the prenatal lethality associated with whole-body homozygous knockouts. The phenotype of these specific mutants could provide additional insights into the precise role of FOXP1 in these neurons.

Third, our study used only male mice for the *in vivo* validation. This cohort selection was necessitated by unanticipated breeding outcomes that yielded a predominantly male population, coupled with the practical constraints of the study’s timeline. This is a notable limitation, considering the significant role of the OXTergic system in female reproductive function. However, since the single-cell computational analysis was performed on a mixed-sex dataset, the identified TFs are more likely to represent a universal developmental program. Consequently, our *in vivo* results in males provide a functional proof-of-concept for a developmental role that appears fundamentally present in the broader population during lineage specification.

Finally, as we did not validate RORA, EBF3, BCL11B, or FOXP2 *in vivo*, we cannot rule out the relevance of these other candidate TFs in differential differentiation. In particular, we may assume that FOXP2 most likely contributes to OXT MCN development as well, considering the effects observed both *in silico* and *in vitro*. A role for FOXP2 could be through FOXP1:FOXP2 heterodimerization, or FOXP2 could function as a redundancy mechanism. Considering the already considerable MCN phenotype of the haploinsufficient *Foxp1*^+/−^ mutant mice, the former seems the more likely option.

## Resource availability

### Lead contact

Further information and requests for resources and reagents should be directed to and will be fulfilled by the corresponding author: Onno Meijer (o.c.meijer@lumc.nl).

### Materials availability

This study did not generate new unique reagents.

### Data and code availability

• scRNA-seq data: The raw data were previously deposited by Romanov et al. (2020).^17^ The processed and annotated developmental mouse MCN dataset has been deposited at Zenodo and is publicly available as of the date of publication. DOIs are listed in the key resources table.
• Code: All original code has been deposited at GitHub (links in KRT) and is publicly available. An archived version of the code has also been deposited to Zenodo. DOIs are listed in the key resources table.
• Any additional information required to reanalyze the data reported in this paper is available from the corresponding contact upon request.

## Acknowledgments

This research was supported by the ZonMw Open Competition grant #09120012010051 (OM, AM), and by the Synergy European Research Council (ERC) grant “OxytocINspace” #101071777 (VG).

## Author contributions

Conceptualization: A.M., P.B., and R.A.; data curation: F.F., H.F., J.B., Q.K., S.T., T.S., and Y.P.; formal analysis: F.F., J.B., Q.K., S.T., T.S., and Y.P.; funding acquisition: A.M., F.A., H.F., O.M., and V.G.; investigation: F.F., J.B., S.T., T.S., and Y.P.; methodology: H.F., J.B., O.M., and V.G.; project administration: J.B.; resources: F.A., O.M., Q.K., and V.G.; software: J.B. and T.S.; supervision: A.M., F.A., O.M., P.B., Q.K., R.A., and V.G.; validation: F.F., H.F., J.B., Q.K., S.T., T.S., and Y.P.; visualization: J.B.; writing – original draft: F.A., H.F., and J.B.; writing – review and editing: A.M., F.A., F.F., O.M., R.A., T.S., and V.G.

## Declaration of interests

The authors declare no conflict of interest.

## STAR★Methods

### Key resources table

**Table:** 

REAGENT or RESOURCE	SOURCE	IDENTIFIER
**Antibodies**		

Mouse anti-Neurophysin 2	Millipore	Cat# MABN856, RRID: AB_3741583
Guinea pig anti-Oxytocin	Synaptic Systems	Cat# 408 004, RRID:AB_2725768
Goat anti-guinea pig Alexa Fluor 488	Thermo Fisher	Cat# A-11073, RRID:AB_2534117
Donkey anti-mouse Alexa Fluor 594	Abcam	Cat# ab150108, RRID:AB_2732073

**Bacterial strains**		

JM109 Competent Cells	Promega	Cat# L2001

**Chemicals, peptides, and recombinant proteins**		

Lipofectamine 2000	Thermo Fisher	Cat# 11668027
M-MLV reverse transcriptase	Promega	Cat# M1705
GoTaq 1-step RT-qPCR master mix	Promega	Cat# A6020
VECTASHIELD Antifade Mounting Medium with DAPI	Vector Labs	Cat# VEC-H-1200

**Critical commerical assays**		

Qiagen Plasmid Midiprep kit	Qiagen	Cat# 12143
Qiagen RNAeasy Micro kit	Qiagen	Cat# 74004

**Deposited data**		

Mouse developmental dataset	Romanov et al.^17^	BioProject: PRJNA548917
Processed MCN dataset	This paper	Zenodo: https://doi.org/10.5281/zenodo.15594917

**Experimental models: cell lines**		

Neuro-2a (N2a)	ATCC	Cat# CCL-131

**Experimental models: Organisms**		

Mouse: Foxp1+/-	Wang et al.^62^	N/A

**Oligonucleotides**		

Primer: EGFP Forward CAC ATG AAG CAG CAC GAC T	This paper	N/A
Primer: EGFP Reverse AGT TCA CCT TGA TGC CGT T	This paper	N/A
Primer: Renilla Forward TCC TTG AGA GTT TTC GCC C	This paper	N/A
Primer: Renilla Reverse CCG GCG TCA ATA CGG GAT A	This paper	N/A

**Recombinant DNA**		

P3.5VPIII.EGFP.IGR2.1 (pVP)	Fields et al.^63^	Addgene #40865
pOTIII.EGFP.IGR3.6 (pOT)	Fields et al.^63^	Addgene #40866
Flag-FOXP1 (pFOXP1)	Moparthi et al.^64^	Addgene #153145
Flag-FOXP2 (pFOXP2)	Moparthi et al.^64^	Addgene #153146
pRL-Renilla (pRL)	Promega	Cat #E2231
pcDNA3.1(+)	Thermo Fisher	Cat #V79020

**Software and algorithms**		

STAR v2.7.11b	Dobin et al.^65^	N/A
scanpy v1.10.2	Wolf et al.^66^	N/A
scvi-tools v1.2.0	Lopez et al.^67^	N/A
QuPath v0.4.3	Bankhead et al.^68^	N/A
Imaris v10.0.1	Oxford Instruments	N/A
scRNA-seq Analysis Code	This paper	https://github.com/jberkh/2025_Avp_Oxt_Diff Zenodo: https://doi.org/10.5281/zenodo.15594917
Cell count Analysis Code	This paper	https://github.com/tim-schubert/impro

### Experimental model and study participant details

#### Mouse models

Mice were kept in a specific pathogen-free Biomedical Animal Facility under a 12-hour light/dark cycle with *ad libitum* access to water and food. All procedures were conducted in strict compliance with the National Institutes of Health Guidelines for the Care and Use of Laboratory Animals and approved by the National Institute of Mental Health animal care and use committee. The day of birth was considered as postnatal day (P) 0.5.

#### Generation of Foxp1^+/-^ animals

WT female mice were crossed with male mice, heterozygous for the *Foxp1* KO allele (*Foxp1*^+/-^).^39^ The *Foxp1*^+/-^ mice were backcrossed with C57BL/6J mice for at least 12 generations to obtain congenic animals.

#### Cell culture

Neuro-2a cells were cultured in Dulbecco’s Modified Eagle Medium (DMEM; Thermo-Fisher Scientific #61965-026) supplemented with 10% fetal bovine serum and 1% penicillin-streptomycin, in a humidified atmosphere, at 37°C and containing 5% CO_2_.

### Method details

#### scRNA-seq data collection and preprocessing

For the mouse developmental dataset from Romanov et al. (2020),^17^ sra data files were obtained from SRA BioProject PRJNA548917, using sra-toolkit 2.11.3. After prefetching the sra files, fastq files were derived with fasterq-dump command, using command-line flags “-p”, “-S”, “--include-technical”. Using the SRA metadata table, fastq files were merged per biological sample. For quantification of the count matrix, STAR^65^ 2.7.11b was used. First, a reference genome was created by running STAR with command-line flags “--runThreadN 20”, “--runMode genomeGenerate”. Files necessary to generate the reference genome were obtained from ENSEMBL. Then, the count matrix was computed for each sample, using STAR with command line flags “--runThreadN 16”, “--runDirPerm All_RWX”, “--readFilesCommand zcat”, “--outSAMtype None”, “--soloType CB_UMI_Simple”, “--soloCBwhitelist 737K-august-2016.txt”, “--soloBarcodeReadLength 0”, “--soloCBlen 16”, “--soloUMIstart 17”, “--soloUMIlen 10”, “--soloStrand Forward”, “--soloUMIdedup 1 MM_CR”, “--soloCBmatchWLtype 1MM_multi_Nbase_pseudocounts”, “--soloUMIfiltering MultiGeneUMI_CR”, “--soloCellFilter EmptyDrops_CR”, “--clipAdapterType CellRanger4”, “--outFilterScoreMin 30”, “--soloFeatures Gene Velocyto”, “--soloMultiMappers EM”, “--outReadsUnmapped Fastx”. Using python 3.11.4, scanpy 1.10.2,^66^ R 4.4.2,^69^ and Seurat 5.1.0,^70^ the count matrices for the different samples were then loaded as single-cell dataset objects, and subsequently merged into a single dataset. Gene names were changes from ENSEMBL gene identifiers to MGI symbols. Finally, using original clustering metadata column “wtree” from Romanov et al. (2020),^17^ a subset was created to include only clusters 13, 14, 15, 16, 24, 26, 31, and 43. Of these, clusters 26 and 43 are the MCN clusters as defined in the original work. To enhance performance of the integration, the transcriptionally related PVN clusters 13-16, 24, and 31 were included in this subset.

#### scRNA-seq data integration

To integrate the mouse developmental dataset, a subset of 1250 most highly variable genes was taken using the function highly_variable_genes, with arguments “n_top_genes = 1250” and “batch_key = sample”. Then, using the scvi-tools 1.2.0 model scVI,^67^ the dataset was integrated on “sample”, using parameters “n_layers = 1”, “n_latent = 10”, “dropout_rate = 0.1”, “dispersion = gene”, “gene_likelihood = “nb”. The model was trained with arguments “max_epoch = 400”, “n_epochs_kl_warmup = 200”, “lr = 1e-2”, “lr_min = 1e-4”, “lr_patience = 33”, “lr_factor = 0.1∗∗(1/3)”, “reduce_lr_on_plateau = True”, “lr_scheduler_metric = elbo_validation”, “check_val_every_n_epoch = 1”, “early_stopping = True”, early_stopping_patience = True”, and “early_stopping_monitor = elbo_validation”.

Subsequently, the trained scVI model was passed to scANVI^18^ and trained again, using the same parameters, except the additional or altered arguments “labels_key = Age”, “unlabeled_category = nan”, “max_epochs = 500”, “classification_ratio = 1.67”. The latent representation of the data was extracted used for downstream processing. Using original clustering metadata column “wtree” from Romanov et al. (2020),^17^ a subset was taken again, this time to only include MCN neurons (clusters 26 and 43).

Since the original ‘wtree’ clustering utilized whole-hypothalamus data, it lacked the granularity required to accurately resolve subtle differences within this highly specific subset. Specifically, the original clustering could not resolve the transcriptomically distinct progenitor cluster, and misassigned OXTergic neurons as AVPergic. As such, we re-clustered the dataset using the subset-specific integrated embeddings. First, neighborhoods were calculated with the FindNeighbors function, using the specified arguments “reduction = vae” and “dim = 1:10”. Subsequently, the dataset was partitioned using the FindClusters and FindSubClusters functions, with resolution parameters set to 0.75 and 0.5, respectively. Finally, the resultant clusters were annotated, and dataset was visualized with the RunUMAP function, using same arguments as for neighborhood calculations.

#### Gene regulatory network inference

Gene regulatory network activities were inferred with the pySCENIC pipeline. The pipeline was run with mostly default settings. For calculating the coexpression modules, the arboreto.algo.grnboost2 function was used with argument “seed = 0” specified, and the pyscenic.utils.modules_from_adjacencies function with argument “keep_only_activating = False” specified. Subsequently, for pruning the modules, pyscenic.prune.prune2df was used with arguments “rank_threshold = 1500”, “auc_threshold = 0.05” and “nes_threshold = 2.0” specified. The regulons were derived with the pyscenic.prune.df2regulons function, and regulon activity score were calculated with the pyscenic.aucell.aucell function, both with default arguments. For this section, python 3.10.13 was used with pyscenic 0.12.1.^19^

#### Cell fate probability inference

To calculate cell fate probabilities, we used 3 modalities: RNA velocity, pseudotime and real-time using optimal transport analysis. For calculating RNA velocity, first a subset of highly variable genes was selected using function highly_variable_genes, with argument “flavor = seurat_v3”. Then, using VELOVI,^25^ the RNA velocity matrix was calculated with default parameters. The training of the model used the same learning parameters as the scVI and scANVI training. The transition matrix was then extracted for downstream CellRank analysis, using default parameters. For calculating pseudotime, psupertime^26^ was used with regularization parameter “n_params = 30”, and ran with parameter “ordinal_data = Age”. Again, the transition matrix was extracted using default parameters. For the optimal-transport analysis, the moscot^27^ TemporalProblem class was used. The analysis was done with parameters “time_key = age_num”, “epsilon = 1e-3”, “tau_a = 0.95”, and “scale_cost = mean”. Here, the transition matrix was extracted using parameters “threshold = auto”, “self_transitions = all”, “conn_weight = 0.2”, and additional connection parameters “n_neighbors = 30”, “use_rep = X_pca”.

For the downstream CellRank 2^20^ analysis, the transition matrices were added with relative weight assigned: 30% pseudotime kernel, 15% velocity kernel, 55% real-time kernel. The GPCCA estimator was fit using parameters “cluster_key = Cluster”, “n_states = [3,7]”, “n_cells = 20”. Initial states were inferred with the predict_initial_states method. Terminal states were inferred with the predict_terminal_states method, using arguments “method = top_n”, and “n_states = 2”. Finally, fate probabilities were calculated with the compute_fate_probabilities method.

#### Transcription factor binding site prediction

Sequences of the *Avp* and *Oxt* mouse promoters were retrieved from NCBI, using genome assembly GRCm39. From this, the SCR sequences were derived, resulting in the *Avp* SCR sequence “TCAACTATGATTTCCAGGTGACCCTCCAGTCGGCTCACCTCACTGATCGCACAGCACCAATCACTGTGGCAGTGGCTCCTGTCACACGGTGGCCGGTGACAGCCTGATGGCTGGCTCCCCTCCTCCACCACCCTCTGCACTGACAGGCCCACGTGTGTCCCCAGATGCCTGA” and *Oxt* SCR sequence “CCCCTTCCAGGCTGCTTCTCTTTTGAGTTCCAGGTCATTAGCAGAGACGATGACCTTGACCCTAGCCCAGACCCTGCAAATGAAGGGCCTGCCTCTAAACAGCGTGGAACAATTTG”. To predict TFBSs, the JASPAR^28^ position weight matrices (PWMs) used were: MA0071.1 (RORA), MA1637.2 (EBF3), MA0481.4 (FOXP1), MA0593.2 (FOXP2), and MA1989.2 (BCL11B). Using the TFBSTools 1.44.0 function searchSeq, SCR sequences were tested for TFBSs using the respective JASPAR PWMs. P-values computed were adjusted using the Holm-Bonferroni method.

#### Plasmids

All used plasmids are listed in the KRT. Plasmids were grown in competent JM109 *E. coli* bacteria (Promega #L2001) at 37°C, and subsequently isolation using a Qiagen Plasmid Midiprep kit (#12143) following the manufacturer’s protocol.

#### Transfections

Cells were seeded 24 hours before transfection at a density of 39,500 cells cm^-2^. Transfections were performed using Lipofectamine 2000 (Thermo Fisher Scientific #11668027) at a DNA:Lipofectamine ratio of 1:3, following the manufacturer’s protocol. Six technical replicates were used per transfected condition. Cells of all conditions were transfected with 131 ng·cm^-2^ reporter plasmid, for either *Avp* (with pVP) or *Oxt* (with pOT), and co-transfected with pRL at 1 ng·cm^-2^as internal control. Varying per condition, cells were transfected with 5.2 ng·cm^-2^ pFOXP1 and/or 5.2 ng·cm^-2^ pFOXP2, or neither. Total transfected plasmid DNA was controlled for with pcDNA3.1, up to a total of 184 ng·cm^-2^.

#### Real-time quantitative PCR

RNA was extracted from cells 48 hours post-transfection, using the Qiagen RNeasy Micro kit (Qiagen #74004), following manufacturer’s protocol. Briefly, cells were lysed and lysates were loaded on silica membrane columns. Bound nucleic acids were washed, and subsequently DNA was removed by on-column DNAse digestion. RNA was eluted and cDNA was synthesized using M-MLV reverse transcriptase (Promega, #M1705), following manufacturer’s instructions. Finally, quantitative PCR was performed using GoTaq 1-step RT-qPCR master mix (Promega #A6020) on the Bio-Rad CFX96 system. Primes used for the RT-qPCR reactions are listed in the KRT.

#### Brain perfusion and immunohistochemistry

Mice were sacrificed at P10, and brains were fixed overnight at 4°C in 4% paraformaldehyde. The following day, the brains were transferred to a 30% sucrose solution in PBS for cryoprotection. After approximately 48 hours, once the brains had sunk to the bottom of the well plates, they were gently blotted with absorbent paper, wrapped in aluminum foil, and stored at –20°C for at least 24 hours.

Cryosectioning was performed using a Leica cryostat. A total of 36 coronal sections, each 50 μm thick, were collected, starting from the anatomical level where the anterior commissures merged. All immunostaining procedures were carried out at room temperature using the free-floating method in well plates. Sections were first washed three times for 10 minutes each in PBS. They were then incubated for one hour in a blocking solution consisting of PBS with 2.5% normal donkey serum (ab7475), 2.5% normal goat serum (ab7481), and 0.1% Triton X-100. Primary antibody incubation was carried out overnight in the same blocking solution supplemented with mouse anti-Neurophysin 2 (1:1000, MABN856) and guinea pig anti-Oxytocin (1:500, 408 004, Synaptic Systems). Following incubation, sections were washed again three times for 10 minutes in PBS. Secondary antibody incubation was performed for four hours in PBS containing 0.1% Triton X-100, goat anti-guinea pig Alexa Fluor 488 (1:1000, A-11073), and donkey anti-mouse Alexa Fluor 594 (1:1000, ab150108). Sections were then washed three times for 10 minutes in PBS. The stained sections were mounted onto SuperFrost Plus adhesion slides (10149870, Epredia) and left to air-dry. VECTASHIELD Antifade Mounting Medium with DAPI (VEC-H-1200) was applied, and the slides were covered with glass coverslips (thickness no. 1, 101242, Marienfeld). Excess mounting medium was absorbed with paper, and nail hardener was applied around the edges of the coverslips to seal them.

#### Slide scanner image acquisition

All coronal brain sections (50 μm thick) containing the hypothalamic paraventricular (PVN), supraoptic (SON) and accessory nuclei (AN) were systematically collected from Foxp1^+/-^ (n=7) and WT (n=7) mice. Brain sections (22-28 per animal) were immunofluorescence-stained for vasopressin and oxytocin as outlined in the previous section. Using an Olympus SLIDEVIEW VS200 system (Evident Scientific, Tokyo, Japan) equipped with a 20x objective, we acquired z-stacks (z-spacing 2.36 μm) of all ROIs containing AVP and OXT neurons in these sections. The system featured an S-Cite NOVEM 9-channell LED illumination system (Excelitas Technologies Corp., Massachusetts, USA), a fast pentaband filter wheel, and an iDS UI-3200SE-M-GL monochrome camera (IDS Imaging Development Systems GmbH, Obersulm, Germany). A standardized scanning protocol was employed across all samples to ensure consistent exposure and image resolution parameters (16 bit Grayscale, 0.35 μm/pixel, 6.45 ms exposure time at 455 nm for DAPI, 26.77 ms exposure time at 565 nm for Cy3. All scanned images were saved in VSI format for downstream analysis.

#### Quantification AVP and OXT neurons

The image in .vsi format is opened using QuPath version 0.4.3. Brightness and contrast settings are adjusted using the “Min display” and “Max display” values to ensure that no image data is lost. Using the rectangle selection tool, the region of interest is selected, either the PVN or SON. In the case of the SON, two separate files are created to analyze the left and right sides independently. From the “Image” tab, the “Image commands” menu is opened and “Send region to ImageJ” is selected with the following settings: resolution set to 2, inclusion of ROI, inclusion of overlay, and all z-slices selected. Once the region is sent, ImageJ opens, and the image is saved via the “File” menu as a TIFF (.tif) file. The saved TIFF file is then opened in Imaris version 10.0.1. In the display adjustment settings, the red channel (channel 1) is turned off along with either the green (channel 2) or blue (channel 3) channel. Typically, the green channel is left on for initial analysis. The display intensity is manually adjusted to reduce background noise as much as possible, based on visual assessment rather than fixed values. The spot function is selected for cell detection. Spot creation parameters are left at their default settings. When needed, the “segment only a region of interest” option is used to simplify the selection of spots. All other algorithm settings remain unchanged. During the spot detection process, channel 2 (green) is first selected as the source, and the estimated cell diameter is set to 10.00 μm, while all other parameters for spot detection are left at their default values. The optional filtering step for spot selection is generally not used, but when cell density is high, the filter is used intuitively to limit false positives and reduce manual workload. The filter is applied conservatively to ensure only visually confirmed cells are selected. Following automatic detection, the “Edit” function is used to manually verify and adjust spot selection. This step is performed by visual inspection, rotating the 3D image to avoid missing any cells and to minimize human error, as no fixed parameters can be defined for this process. Once all cells are selected, the “Statistics” function is used to generate a count of the detected spots. The data is then exported using the “Export statistics on Tab display to file” function into an Excel (.xls) file. This process is repeated for each image containing a visible PVN, for both sides of the SON, and separately for each recording channel, including repeating the same analysis using the blue channel. All image processing, quantification, and data analysis were conducted entirely blind to experimental conditions.

#### Confocal image acquisition

Representative high-resolution images for visualization purposes were acquired using a confocal microscope (Stellaris 5, Leica Microsystems, Wetzlar, Germany) equipped with a 40x objective (HC PL APO CS2 40×/1.25 GLYC, Leica Microsystems, Wetzlar, Germany). Image stacks were obtained from 50 μm-thick sections using a z-step interval of 1 μm. The acquisition settings were as follows: 1024 × 1024 pixels, 16-bit depth, pixel size 0.38 μm, and a zoom factor of 0.75.

### Quantification and statistical analysis

#### Image analysis and neuron counting

Spot counts from individual Imaris reconstructions were aggregated per animal and cell type using R 4.4.2.^69^

#### Statistics

Statistical tests were performed in R 4.4.2.^69^ P-values smaller than 0.05 were considered statistically significant. For the comparison of the in-vitro reporter assays, a two-way ANOVA was performed with FOXP1, FOXP2, and the interaction term as independent variables. The post-hoc analysis was performed using Tukey’s Honest Significant Difference test. For the comparisons of neuron counts and OXT/AVP ratios between *Foxp1*^+/-^ and WT mice, a Welch’s t-test was performed.
